# Combined Ipsilateral Humeral Shaft and Galeazzi Fractures Creating a Floating Elbow Variant

**DOI:** 10.1155/2018/7430297

**Published:** 2018-11-08

**Authors:** Patrick Lee, Allison Z. Piatek, Michael J. DeRogatis, Paul S. Issack

**Affiliations:** Department of Orthopaedic Surgery, New York-Presbyterian Hospital, New York, USA

## Abstract

“Floating elbow” injuries of the arm traditionally represent a combination of humeral shaft and forearm fractures which require anatomic rigid open reduction and internal fixation of all fractures to allow for early range of motion exercises of the elbow. There are published variants of the floating elbow injury which include ipsilateral diaphyseal humeral fracture, proximal ulna fracture with proximal radioulnar joint disruption, and ipsilateral diaphyseal humeral fracture with elbow dislocation and both bones forearm fracture. We present the case of a 21-year-old woman whose left arm became caught between the side of a waterslide and adjacent rocks at a park. She sustained a torsional and axial loading injury to her left upper extremity resulting in ipsilateral humeral shaft and Galeazzi fractures. The combination of ipsilateral humeral shaft and Galeazzi fractures resulted in a rare floating elbow variant. Prompt open reduction and internal fixation of both fractures and early range of motion of the elbow and wrist resulted in an excellent clinical and radiographic result. Floating elbow injuries and their variants should be promptly recognized as early anatomic reduction, and rigid internal fixation can allow for good elbow function with minimization of stiffness.

## 1. Introduction

The combination of humeral shaft and forearm fractures results in a “floating elbow” injury which requires open reduction and internal fixation of all fractures to allow for preservation of elbow joint motion and minimization of stiffness [1–3]. Variants have been described which include ipsilateral diaphyseal humeral fracture, proximal ulna fracture with proximal radioulnar joint disruption (Monteggia fracture), and ipsilateral diaphyseal humeral fracture with elbow dislocation and both bones forearm fracture [4–8]. We describe here a case of a patient who sustained an ipsilateral humeral shaft fracture and Galeazzi fracture from a torsional and axial loading injury to her upper extremity. This is a rare combination of injuries which creates a floating elbow variant with disruption of the distal radioulnar joint [9]. The patient was informed that the data concerning the case would be submitted for publication, and the patient agreed.

## 2. Case Report

**Figure 1:**
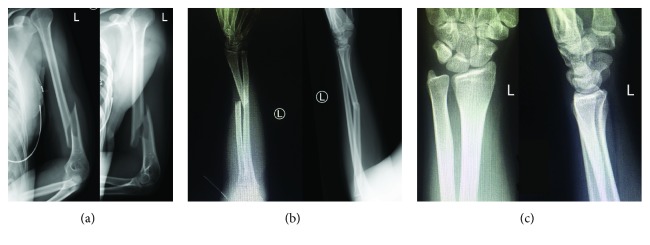
Anteroposterior (AP) and lateral radiographs of the humerus (a), forearm (b), and wrist (c) demonstrating a displaced left distal third humeral shaft fracture, a displaced left midshaft radius fracture with dislocation of the left distal radioulnar joint.

A 21-year-old previously healthy woman, a college student, was on a waterslide at an amusement park, when her left arm became caught between the side of the waterslide and adjacent rocks. She sustained a forceful twisting and loading injury to her left upper extremity which resulted in a severe pain and deformity to her left arm, forearm, and wrist. She was brought by ambulance to our emergency department. On physical exam, her injuries were closed, her arm and forearm compartments were soft, and she was neurovascularly intact. Radiographs of the humerus, forearm, and wrist demonstrated a left distal third humeral shaft fracture (Figure 1(a)) as well as a left Galeazzi fracture, with a midshaft radius fracture and disruption of the distal radioulnar joint (Figures 1(b) and 1(c)). She was placed in a well-padded long arm posterior plaster splint extending from the posterior shoulder to the fingers, with an additional coaptation component to the splint to stabilize the humerus fracture.

Within 24 hours of admission, the patient underwent open reduction and internal fixation of both her humeral shaft and radial shaft fractures. The decision was made to reduce and fix the humeral shaft first. The humeral shaft fracture was amenable to reduction and fixation with an extraarticular locking plate through a posterior approach with the patient in the lateral decubitus position. Because of the manipulation of the forearm, which may be required during reduction and plating of the humerus, we chose to avoid fixation of the radius and the possible transfixion of the distal radioulnar joint until after the operation on the humeral shaft was completed.

**Figure 2:**
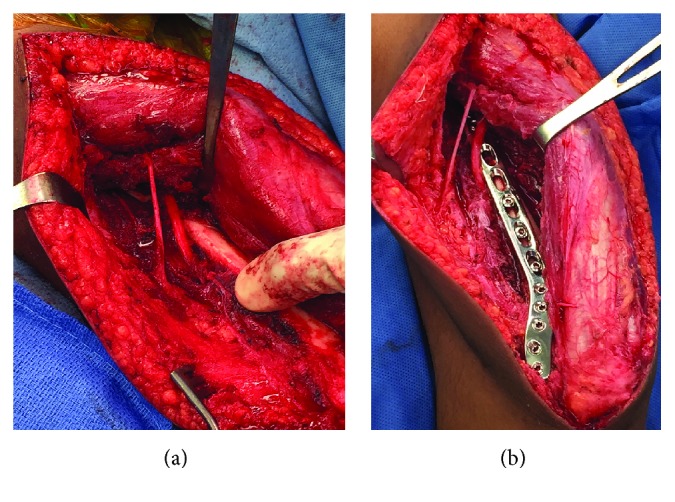
Exposure of the posterior humerus through the Gerwin-Hotchkiss approach. (a) Intraoperative photograph of surgical exposure with retraction of the triceps mechanism medially demonstrating the humeral fracture site and the radial nerve in the spiral groove. (b) Intraoperative photograph after open reduction and internal fixation of the humeral shaft using an extraarticular locking plate.

The surgery was performed under general anesthesia. The patient was positioned on a radiolucent table in the lateral decubitus position with the left arm which was supported over a foam roller. The left upper extremity was then prepped and draped, and 2 grams of cefazolin was administered prior to incision. The Gerwin-Hotchkiss approach was used to access the posterior half of the distal humerus and radial nerve [10]. A longitudinal incision was made in the midline of the posterior aspect of the arm, and the deep fascia of the arm was incised. After identification and protection of the ulna nerve, the entire triceps mechanism was retracted medially to identify the main trunk of the radial nerve proximal to its point of entry deep to the intermuscular septum. The intermuscular septum was divided distally approximately 3 centimeters, and then, the medial and lateral heads of the triceps muscle were then mobilized from lateral to medial along with the radial nerve to expose the posterior humeral shaft (Figure 2(a)). After reduction, a 3.5 mm extraarticular distal humerus plate (Depuy Synthes, West Chester, PA) was used for humerus fixation (Figure 2(b)). After fluoroscopic confirmation of reduction and fixation, the wound was closed and dressed. The patient was then positioned supine on the radiolucent table with the left arm extended over a hand table attachment. The volar Henry approach was performed to access the radial shaft fracture. The fracture was anatomically reduced and plated using a prebent 3.5 limited contact dynamic compression plate. Anatomic reduction of the radius with restoration of the radial bow reduced the distal radioulnar joint. The distal radioulnar joint remained reduced and stable through full pronation and supination. Therefore, an open reduction was not indicated. Subcutaneous tissue and skin were closed, and a dry sterile dressing with well-padded long arm splint was applied.

**Figure 3:**
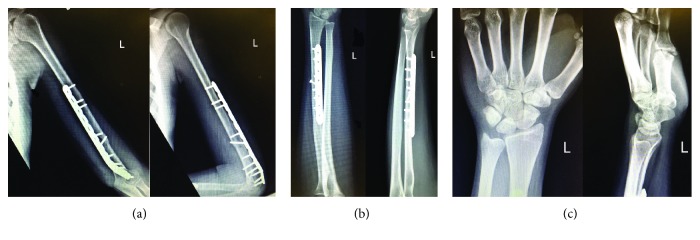
One year postoperative AP and lateral radiographs of the left humerus (a), forearm (b), and wrist (c) demonstrating healing of both humeral and radial shaft fractures and maintenance of reduction of the distal radioulnar joint.

**Figure 4:**
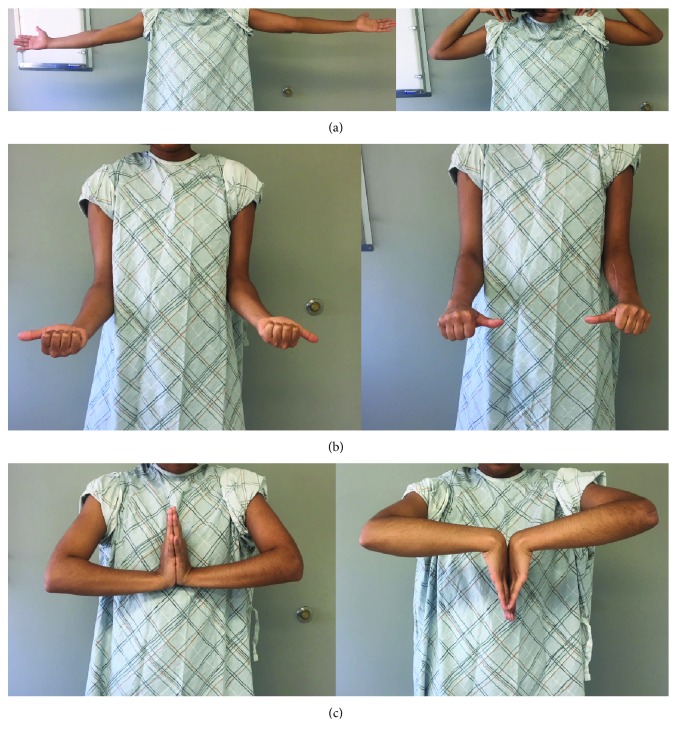
Clinical photographs of the patient at 1 year postoperatively demonstrating elbow flexion and extension (a), forearm pronation and supination (b), and wrist flexion and extension (c).

The patient's postoperative course was unremarkable. The splint was removed at one week, and gentle elbow and wrist active and passive range of motion exercises were begun. At 12 weeks follow-up, radiographs demonstrated healing of the humeral and radial shaft fractures. At this time, elbow, forearm, and wrist strengthening exercises were performed for 6 weeks. At final follow-up 1 year postoperatively, radiographs demonstrated healed humeral and radial shaft fractures in anatomic position with stable reduction of the distal radioulnar joint (Figures 3(a)–3(c)). The patient is pain-free and has full range of motion (0–135 degrees flexion, 90 degrees of pronation and supination, and 85 degrees of wrist flexion and extension) and excellent clinical function (Figures 4(a)–4(c)).

## 3. Discussion

Floating elbow injuries traditionally refer to ipsilateral diaphyseal humeral fracture and diaphyseal ulna and radius fractures [2, 3]. These are significant injuries with potential for complications including stiffness, neurovascular injury, open wounds, and compartment syndrome [2, 3, 5, 7, 11]. Anatomic reduction and internal fixation of these fractures with early elbow range of motion exercises can maintain a functional range of motion [2, 3, 5, 7].

There are few series which evaluate the outcomes of operative treatment of floating elbow injuries. All of the patients in earlier case series had traditional floating elbow injuries with diaphyseal fractures of the humerus ulna and radius [2, 3]. Yokoyama and colleagues retrospectively reviewed 15 floating elbow injuries with diaphyseal fractures of the humerus, radius, and ulna treated with open reduction and internal fixation. At a mean follow-up of 43 months, the mean arc of elbow motion was 105.7 ± 31.8° [3]. Solomon and colleagues retrospectively reviewed 18 patients with traditional floating elbow injuries treated with open reduction and internal fixation. Average elbow motion was 17° to 115° [2].

More recent case series have included floating elbow variants including ipsilateral diaphyseal humeral fracture with proximal ulna fracture, and proximal radioulnar joint disruption (Monteggia fracture); ipsilateral diaphyseal humeral fracture, elbow dislocation, and diaphyseal ulna and radius fracture; and distal humerus fractures with intraarticular fractures of the olecranon or radial head [5, 7]. Jockel and colleagues retrospectively reviewed 19 patients with floating elbow injuries with variants of the traditional injury including 5 patients with an ipsilateral diaphyseal humeral fracture, proximal ulna fracture, and proximal radioulnar joint disruption (Monteggia fracture) and 2 patients with an ipsilateral diaphyseal humeral fracture, elbow dislocation, and diaphyseal ulna and radius fractures. At a mean follow-up of 6.7 years after open reduction and internal fixation, the mean American Shoulder and Elbow Society elbow score was 89 (range, 13–99). Mean elbow range of motion was 10° to 126°. Complications included 4 nonunions [7].

Ditsios and colleagues retrospectively reviewed 19 patients with floating elbow injuries and reported variants in their series including humerus shaft or distal humerus fractures and an intra-articular fracture of the olecranon and/or the radial head. They included three patients with humeral shaft and Galeazzi fractures and classified them as traditional fracture patterns. Two of these cases were distal third humeral shaft fractures (Holstein-Lewis pattern) with radial nerve palsy. At a mean follow-up of 26 months, the final arc of motion in the entire cohort was 88.9° (range, 30°–110°), and 11 patients (58%) had good-excellent results. Because the three cases of humeral shaft with Galeazzi fracture were not separately analyzed, it is unclear if the results of this subgroup are different from those of the traditional floating elbow pattern [5]. Other than these three cases, there is one case report describing a patient with a humeral shaft, Galeazzi fracture and elbow dislocation. That patient was treated first with closed reduction of the elbow joint followed by intramedullary nailing of the humerus and plating of the radius, and obtained full functional range of motion of the elbow, forearm, and wrist at 5 months postoperatively [8].

We report here a very rare floating elbow variant with fracture of the humeral shaft, fracture of the radial shaft, and distal radioulnar joint disruption (Galeazzi fracture). Ipsilateral humeral shaft and Galeazzi fracture is a floating elbow pattern that should be recognized as an atypical pattern distinct from the traditional pattern in which the wrist joint is intact. Disruption of the distal radioulnar joint and the potential effect of this injury on wrist function adds an additional variable not present with the traditional fracture type that must be addressed in order to obtain good functional outcomes.

## References

[1] Jimenez-Diaz V., Aunon-Martin I., Olaya-Gonzalez C., Aroca-Peinado M., Cecilia-Lopez D., Caba-Doussoux P. (2017). Analysis of complications after a floating elbow injury. *European Journal of Orthopaedic Surgery and Traumatology*.

[2] Solomon H. B., Zadnik M., Eglseder W. A. (2003). A review of outcomes in 18 patients with floating elbow. *Journal of Orthopaedic Trauma*.

[3] Yokoyama K., Itoman M., Kobayashi A., Shindo M., Futami T. (1998). Functional outcomes of “floating elbow” injuries in adult patients. *Journal of Orthopaedic Trauma*.

[4] De Carli P., Boretto J. G., Bourgeois W. O., Gallucci G. L. (2006). Floating dislocated elbow: a variant with articular fracture of the humerus. *The Journal of Trauma*.

[5] Ditsios K., Boutsiadis A., Papadopoulos P. (2013). Floating elbow injuries in adults: prognostic factors affecting clinical outcomes. *Journal of Shoulder and Elbow Surgery*.

[6] Galasso O., Mariconda M., Gasparini G. (2011). Repeated floating elbow injury after high-energy trauma. *Strategies in Trauma and Limb Reconstruction*.

[7] Jockel C. R., Gardenal R. M., Chen N. C., Golden R. D., Jupiter J. B., Capomassi M. (2013). Intermediate-term outcomes for floating elbow and floating elbow variant injuries. *Journal of Shoulder and Elbow Surgery*.

[8] Sarup S., Bryant P. A. (1997). Ipsilateral humeral shaft and Galeazzi fractures with a posterolateral dislocation of the elbow: a variant of the “floating dislocated elbow”. *The Journal of Trauma: Injury, Infection, and Critical Care*.

[9] Atesok K. I., Jupiter J. B., Weiss A.-P. C. (2011). Galeazzi fracture. *The Journal of the American Academy of Orthopaedic Surgeons*.

[10] Gerwin M., Hotchkiss R. N., Weiland A. J. (1996). Alternative operative exposures of the posterior aspect of the humeral diaphysis with reference to the radial nerve. *The Journal of Bone and Joint Surgery-American Volume*.

[11] Ring D., Waters P. M., Hotchkiss R. N., Kasser J. R. (2001). Pediatric floating elbow. *Journal of Pediatric Orthopedics*.

